# An Attempt to Explain the Nature of Electricity and Its Intention in the Economy of the Universe

**Published:** 1844-10

**Authors:** 


Art. XIII.—*An Attempt to Explain the Nature of Electricity and its intention in the Economy of the Universe.* By Robert Serrell Wood. Philadelphia: Charles F. Town printer. 1844. 8vo. pp. 90.

Here is a great attempt—no less than that of explaining the *Nature* of *Electricity.* The paper was laid before the National Institute at its last meeting, and the author claims for his views decided originality, as well as plausibility for his arguments. We hope the members of the Institute have been more successful in discovering the former, as well as more forcibly struck with the latter, than we were with these boasted qualities in a book which we lately reviewed on the *Curability of Consumption.* It is not every author who has the happiness to be understood, if to be comprehended, and to have ones claims admitted are the same thing. Many, now a days, as in the days of Pope, may be heard complaining—

"Some read, none praise, and few understand."

We confess, we have not read Mr. Robert Serrell Wood's pamphlet further than his table of contents, and are, therefore, not prepared to praise it, as we do not pretend to understand it. This, however, we will promise,—to submit it to a friend who has a taste for such abstruse speculations, and, to lay before our readers the result of his critical labors. Meanwhile, they will rejoice in the fruitful showers which this electricity collects and disperses over the land, although unable to explain its nature. Y.

